# Application of a model‐based water‐equivalent EPID image conversion algorithm for linac beam QA

**DOI:** 10.1002/acm2.70210

**Published:** 2025-08-15

**Authors:** Ivan Kutuzov, Boyd McCurdy

**Affiliations:** ^1^ Department of Physics and Astronomy University of Manitoba Winnipeg Manitoba Canada; ^2^ Medical Physics Department CancerCare Manitoba Winnipeg Manitoba Canada; ^3^ Department of Radiology University of Manitoba Winnipeg Manitoba Canada

**Keywords:** EPID, EPID dosimetry, linac QA, machine QA, water‐equivalent EPID

## Abstract

**Purpose:**

The nonwater‐equivalent energy response of electronic portal imaging devices (EPIDs) is a major obstacle to using them for linear accelerator (linac) beam parameter verification. In this study, we propose an EPID‐based machine quality assurance (QA) application that uses a model‐based radiation transport algorithm to convert EPID‐measured images into water‐equivalent dose distributions that can be used to assess beam flatness and symmetry.

**Methods:**

An in‐house developed, model‐based radiation transport algorithm was used to estimate the incident beam fluence from measured EPID images and convert it into either 3D dose distributions in a virtual water tank or 2D water‐equivalent dose distributions in a virtual ion chamber array. The conversion algorithm was validated using independent measurements in a scanning water tank and a reference ion chamber array under symmetric and also intentionally detuned (i.e., asymmetric) beam conditions.

**Results:**

For symmetric fields, EPID‐reconstructed percentage depth dose distributions (PDDs) agreed with water tank measurements to within 1% beyond the first 10 mm of depth. Beam profile comparisons showed differences within 1% in low dose‐gradient regions. For all symmetric and intentionally asymmetric fields, beam flatness and symmetry derived from reconstructed images agreed with reference measurements to within 0.2% and 0.3%, respectively. The model demonstrated high sensitivity to the controlled beam asymmetries and steering distortions, with EPID‐reconstructed metrics closely matching reference water‐equivalent measurements and significantly outperforming metrics derived from raw EPID images.

**Conclusions:**

The proposed model‐based algorithm enables accurate conversion of EPID images into water‐equivalent dose distributions, facilitating accurate determination of beam flatness and symmetry. This application addresses some limitations of the previously proposed EPID‐based linac QA techniques, which are limited to nonwater‐equivalent constancy checks, and supports the use of EPIDs as robust dosimetry tools for linac radiation beam parameter verification.

## 1 INTRODUCTION

Recently, there has been growing interest in EPID‐based machine QA in radiotherapy, motivated by the high reproducibility of amorphous silicon (a‐Si) EPID detectors and their convenience, which allows users to save time and resources.^1^ EPIDs also demonstrate favorable dosimetric characteristics such as fast deployment, digital image acquisition, large detector area, high spatial and temporal resolution, linear dose and dose rate response, and favorable long‐term stability.^1^, ^2^, ^3^ In addition, EPIDs are included on nearly all modern linacs.^1^

The majority of EPID‐based machine QA applications proposed in the literature to date have focused on verifying geometric and mechanical linac parameters.^4^, ^5^, ^6^, ^7^, ^8^, ^9^, ^10^ In particular, there have been publications on the use of EPID for determining jaw positioning and field size,^4^ light and radiation field coincidence,^5^ gantry and collimator sag,^6^ multi‐leaf collimator (MLC) positioning accuracy^7^, ^8^ and leaf speed,^8^ and for machine isocentricity checks.^9^, ^10^ There have also been proposals to use EPID for checking constancy of radiation beam parameters such as beam energy,^11^, ^12^, ^13^ machine output,^12^, ^13^, ^14^, ^15^ beam flatness^12^, ^13^, ^14^, ^15^, ^16^ and symmetry.^12^, ^13^, ^14^, ^15^, ^16^, ^17^ At the time of writing this manuscript, AAPM Task Group No. 330 is developing comprehensive guidelines and recommendations for using EPIDs for linac QA, including both in‐house developed and commercially available EPID‐based machine QA applications.^18^

The AAPM Task Group No. 142 guidelines require key linac radiation beam parameters—such as flatness, symmetry, beam energy, and output—to be measured in water or a water‐equivalent medium at specified depths.^19^, ^20^ However, a‐Si EPIDs are nonwater‐equivalent detectors that over‐respond to low energy photons compared to water‐equivalent detectors^15^, ^16^, ^17^ and have inherent radiological depth differences from those specified for linac QA measurements.^21^ In addition, the flood field correction typically applied by the EPID's image acquisition software removes the impact of the incident beam fluence profile from the reported images, resulting in a loss of valuable dosimetric information.^15^ The proposed EPID‐based techniques for monitoring photon beam parameters^12^, ^13^, ^14^, ^15^, ^16^, ^17^ rely on measured EPID images without accounting for the detector energy response and radiological depth. This limits the use of these EPID‐based machine QA applications to constancy checks relative to the baselines determined at the time of reference measurements.^15^, ^17^ While constancy measurements are helpful, absolute beam profile parameters such as flatness and symmetry, cannot be determined using these applications,^15^ and there is clear benefit if the EPID could provide water‐equivalent dosimetry.

There have been a number of empirical algorithms published in the literature that convert nonwater‐equivalent EPID images (dose‐to‐phosphor) into water‐equivalent images (dose‐to‐water).^22^, ^23^, ^24^, ^25^, ^26^, ^27^, ^28^ These can potentially be used to address the nonwater‐equivalent response of a‐Si EPID detectors and facilitate an accurate definition of beam profile parameters (e.g., absolute beam flatness and symmetry) using EPID‐measured images. However, most published studies do not apply the proposed conversion algorithms for specific linac QA applications but rather offer them as a general image conversion tool^24^, ^25^, ^26^, ^27^, ^28^ or as a patient‐specific intensity‐modulated radiotherapy (IMRT) QA tool.^22^, ^23^

In particular, King et al. developed an empirical model to derive the photon energy fluence incident on the EPID from a measured image and convert the fluence to the dose that would be deposited in a water tank at a given depth.^22^ The dose model was optimized for pretreatment IMRT verification. The authors pointed out that it should be possible to use the developed dose model for routine linac QA, however, this possibility has not been explored.^22^ Miri et al. reported on an in‐house developed model to convert EPID images to a planar dose in a virtual water phantom.^23^ The model performance was investigated for IMRT QA applications and validated for a variety of incident open fields at several specified depths. However, the study did not focus on the determination of linac beam parameters, and the proposed model was not evaluated using detuned incident beams.^23^

Correction‐based algorithms that rely on empirical or semi‐empirical conversion functions may lack robustness when it comes to varying measurement geometries.^29^ Correction functions may have to be re‐interpolated every time users need to change parameters such as measurement depth in water^22^, ^23^, ^24^ or source‐detector distance (SDD).^25^ This makes it difficult for such algorithms to maintain dose calculation accuracy over a range of measurement scenarios. In contrast, model‐based techniques tend to be significantly more robust and accurate over a wider range of geometries compared to correction‐based methods.^29^

In this study, we propose an EPID‐based machine QA application that utilizes a previously developed model‐based radiation transport algorithm.^30^, ^31^, ^32^, ^33^, ^34^, ^35^ This algorithm was recently modified to convert EPID‐measured nonwater‐equivalent images into either (1) corresponding 2D water‐equivalent images in a virtual ion chamber array, or (2) into 3D dose distributions in a virtual water tank.^36^, ^37^ The derived water‐equivalent dose distributions generated in a virtual water‐equivalent detector or phantom can subsequently be used for accurately determining linac radiation beam parameters, such as beam flatness and symmetry. The terms “EPID reconstructed” and “virtual” phantom will be used in this work to describe the output of the conversion of raw EPID images to water‐equivalent dose.

Another potential use of the developed application is detecting changes in beam energy. However, this requires a significant separate investigation to assess the method's sensitivity and accuracy and is therefore not included in the current study. The proposed application to beam flatness and symmetry is tested here for a variety of incident fields by introducing various levels of controlled beam asymmetry or “detuning”, and its accuracy and sensitivity in converting the EPID measurements to a virtual water estimate are validated using independent reference measurements in a water‐equivalent ion chamber array and a water tank.

## 2 METHODS

### 2.1 Model‐based, water‐equivalent dose conversion algorithm

**FIGURE 1:**
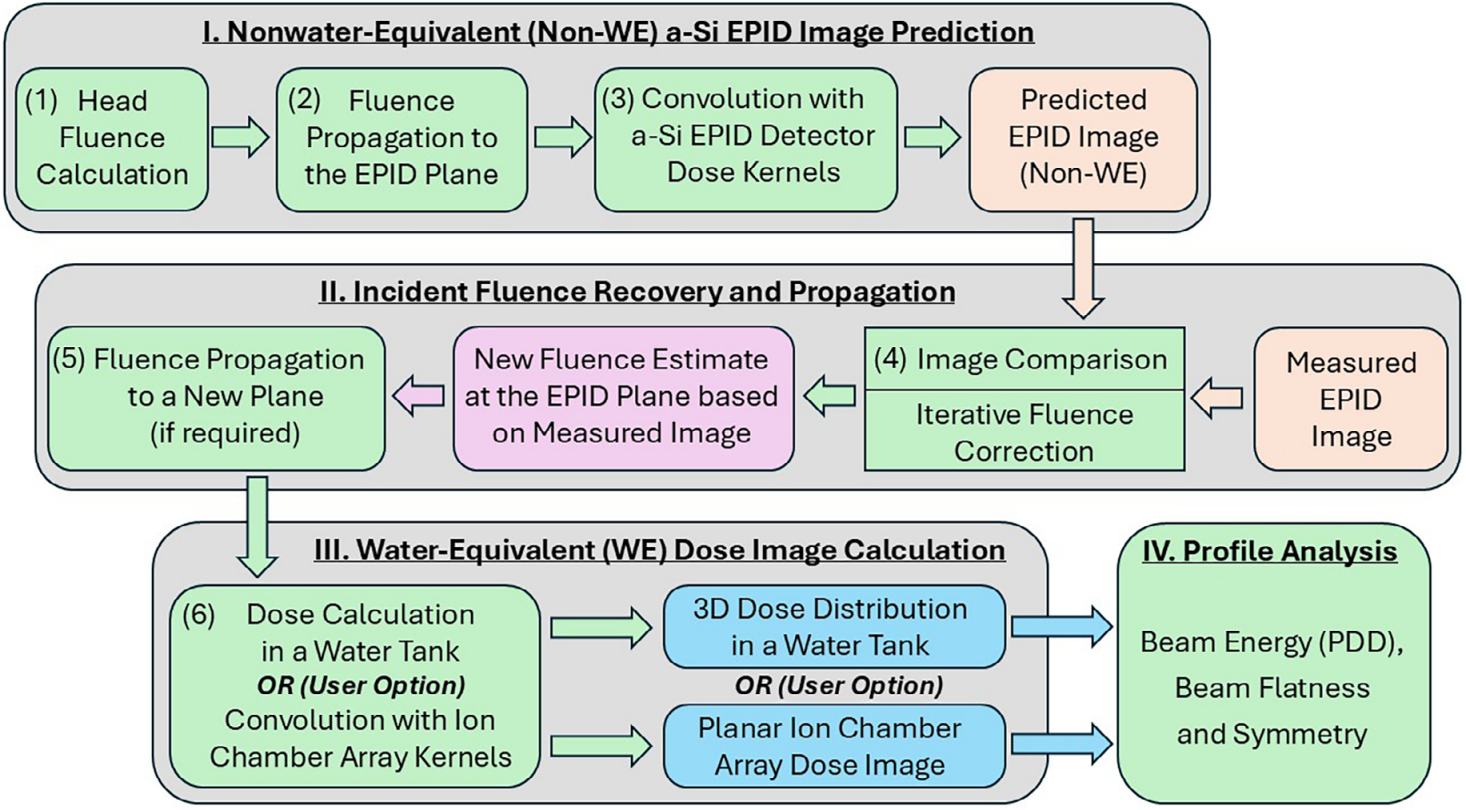
Workflow of the model‐based water‐equivalent EPID image conversion algorithm. Step 4 is an iterative algorithm to estimate the incident fluence from the measured EPID image. 10 × 10 cm^2^ and 20 × 20 cm^2^ open fields measured at 100 cm SDD were used for model validation.

The model‐based water‐equivalent EPID image conversion algorithm used in this study is based on an in‐house developed comprehensive linac radiation transport model^30^, ^31^, ^32^, ^33^, ^34^, ^35^, ^36^, ^37^ previously tested for in vivo dosimetry applications^32^, ^33^, ^34^ and patient‐specific pretreatment QA.^35^ Recently this model was extended to use water‐equivalent detectors systems^36^, ^37^ and verified to show that EPID measured images could be accurately converted to 2D water equivalent images^36^ or used to reconstruct dose in a 3D water tank.^37^ The input data required by the algorithm is the measured a‐Si EPID image to be converted into a water‐equivalent image, the field parameters related to that measured image (e.g., jaw and MLC leaf positions, SDD, etc.), the new SDD (if different from the original measured EPID image), and whether a 2D or 3D dose distribution is required as output. The implemented model workflow is demonstrated in Figure 1 and is briefly summarized below.

In the first step, a predicted EPID image is calculated using the same beam configuration as a treatment plan that was used to deliver a test QA field and measure a corresponding EPID image. The linac head fluence is calculated using a two‐source fluence model, which has been described in detail previously.^30^, ^31^ The calculated head fluence estimate is transported to the EPID plane, accounting for the inverse square effect, and then that fluence estimate is convolved with the detector‐specific dose deposition kernels to calculate the predicted EPID image.^30^, ^31^

In the second step, the predicted EPID image is compared to the actual measured image, and an iterative fluence correction algorithm is implemented, as described in earlier publications.^32^, ^33^ Dark field correction and pixel sensitivity correction are applied to the EPID‐measured image. Flood field correction is not applied, in order to retain the incident fluence profile in the measured image. Adjustments are iteratively made in the predicted fluence until the corresponding predicted EPID image matches the measured EPID image. This results in an accurate incident fluence estimate entering the EPID plane, based on the actual measurement.

In the third step, the incident fluence estimate derived from the measured EPID image is used to obtain a corresponding water‐equivalent dose image^36^ or 3D dose distribution in a water tank,^37^ at a user defined SDD. A reconstructed water tank dose distribution could be used for a beam quality check using PDD (although, this application is not explicitly studied here), and the beam profile can be used to determine beam flatness and symmetry according to the AAPM guidelines.^19^, ^20^ Alternatively, a water‐equivalent, 2D ion chamber array image can be used to determine beam flatness and symmetry.^19^, ^20^ In summary, the model can calculate either 2D or 3D water‐equivalent dose distributions based on the incident fluence estimated from a measured EPID image.

Equipment used in this study included a clinically used TrueBeam machine (Varian Medical Systems, Palo Alto, USA) equipped with an aS1200 EPID detector. Regular (i.e., nonwater‐equivalent) EPID images were acquired using this detector and then converted into either 3D dose distributions in water or water‐equivalent planar images using the above‐described algorithm. Independent reference 3D dose distributions in a water tank were made using the MP3‐T water phantom system and analyzed using MEPHYSTO software (both PTW Dosimetry, Freiburg, Germany). Independent reference planar measurements were made using a MatriXX Evolution ionization chamber array and analyzed using OmniPro software (both IBA Dosimetry, Schwarzenbruck, Germany).

### 2.2 Validation of the EPID‐to‐water conversion algorithm using independent scanning water tank measurements

Two open square field EPID images were acquired at a 100 cm SDD using dosimetry acquisition mode and a flattened 6MV beam at 600 MU/min dose rate. The field sizes were 10 × 10 cm^2^ and 20 × 20 cm^2^ at isocenter, and the amount of delivered fluence was 100 MU for each field size. The images were then converted into water‐equivalent 3D dose distributions in a virtual 40 × 40 × 40 cm^3^ water tank at a 100 cm source‐to‐surface distance (SSD) using the above‐described algorithm^37^ presented in Figure 1. The resultant water‐equivalent 3D dose distributions were compared against the independent reference measurements in a scanning water tank. The compared beam parameters included PDDs, crossplane and inplane profiles at d_max_ (14 mm for 10 × 10 cm^2^ field and 12 mm for 20 × 20 cm^2^ field) and 10 cm depth, and beam flatness and symmetry values calculated at the same depths.

Beam flatness and symmetry were determined from the beam central axis (CAX) profiles using the following definitions^13^:

(1) $$\mathrm{Flatness}=\frac{M-m}{M+m}\;\times 100$$

(2) $$\mathrm{Symmetry}=\frac{\max\left(D_{left}-D_{right}\right)}{D_{center}}\;\times 100$$

where M and m are the maximum and minimum beam intensity values within the central 80% of the profile width, $D_{left}$ and $D_{right}$ are the beam intensity values from two equidistant points from the central axis within the central 80% of the profile width, and $D_{center}$ is the beam intensity value at the central axis.

### 2.3 Assessment of model sensitivity using asymmetrical open fields

The assessment of model sensitivity to incident fluence variations was made using open field measurements with controlled beam symmetry distortions introduced. A series of EPID measurements was made at 100 cm SDD using dosimetry acquisition mode and a flattened 6MV beam at 600 MU/min dose rate. First, an undistorted 20 × 20 cm^2^ open field was measured using 500 MU to serve as a baseline reference. Subsequent images in the series were acquired by repeating a 500 MU, 20 × 20 cm^2^ open field and then delivering an additional 5, 10, 15, or 20 MU using a half‐blocked 10 × 20 cm^2^ field on either side of the beam central axis. This resulted in two sets of nine images that gradually skewed asymmetry to each side, with one set in the crossplane direction and one set in the inplane direction. Each set of images contained open field profiles with estimated symmetry distortions of approximately 0%, ± 1%, ± 2%, ± 3%, ± 4%, including the baseline image denoted here as having zero percent distortion.

The EPID‐measured images were then converted into water‐equivalent 2D dose distributions in a virtual MatriXX ion chamber array with a 5 cm solid water buildup layer, at a 100 cm SDD using the above‐described algorithm.^36^, ^37^ The beam profiles orthogonal to the CAX of the resultant water‐equivalent images were compared against the independent reference measurements in a MatriXX ion chamber array with a 5 cm solid water buildup acquired at the same beam settings and SDD as the EPID measurements. Beam flatness and symmetry were calculated for each image using Equations (1) and (2) and the EPID‐reconstructed water‐equivalent images were compared with the MatriXX‐measured images.

### 2.4 Validation of model accuracy using beam de‐steering

The model performance was also investigated for situations of modified beam steering. This experiment used asymmetric beams detuned in the inplane and crossplane directions by adjusting the radial and transverse angles of beam steering. A series of six EPID measurements was acquired at a 100 cm SDD using dosimetry acquisition mode and a flattened 6MV beam at 600 MU/min dose rate. All measurements were taken using a 20 × 20 cm^2^ open field at isocenter, with 500 MU delivered for each measurement. Reference MatriXX measurements were acquired with a 5 cm solid water buildup after each EPID measurement with the same beam parameters. The first measurement with no beam steering adjustment served as the baseline reference for this experiment. For the next four measurements, the beam was detuned in either inplane or crossplane direction by manually changing the radial or transverse beam steering angle. For the last, sixth measurement, the beam was detuned in both directions.

The images were converted into corresponding water‐equivalent 2D dose distributions in a virtual MatriXX ion chamber array with a 5 cm solid water buildup, at a 100 cm SDD using the same algorithm described in the previous section (Figure 1). Then the beam flatness and symmetry were calculated using Equations (1) and (2) for each test image. The EPID‐reconstructed water‐equivalent images and MatriXX‐measured water‐equivalent images were compared to validate the accuracy of the proposed water‐equivalent image conversion algorithm and its ability to estimate derived water‐equivalent beam parameters.

## 3 RESULTS

### 3.1 Validation of the EPID‐to‐water image conversion algorithm using independent scanning water tank measurements

**FIGURE 2:**
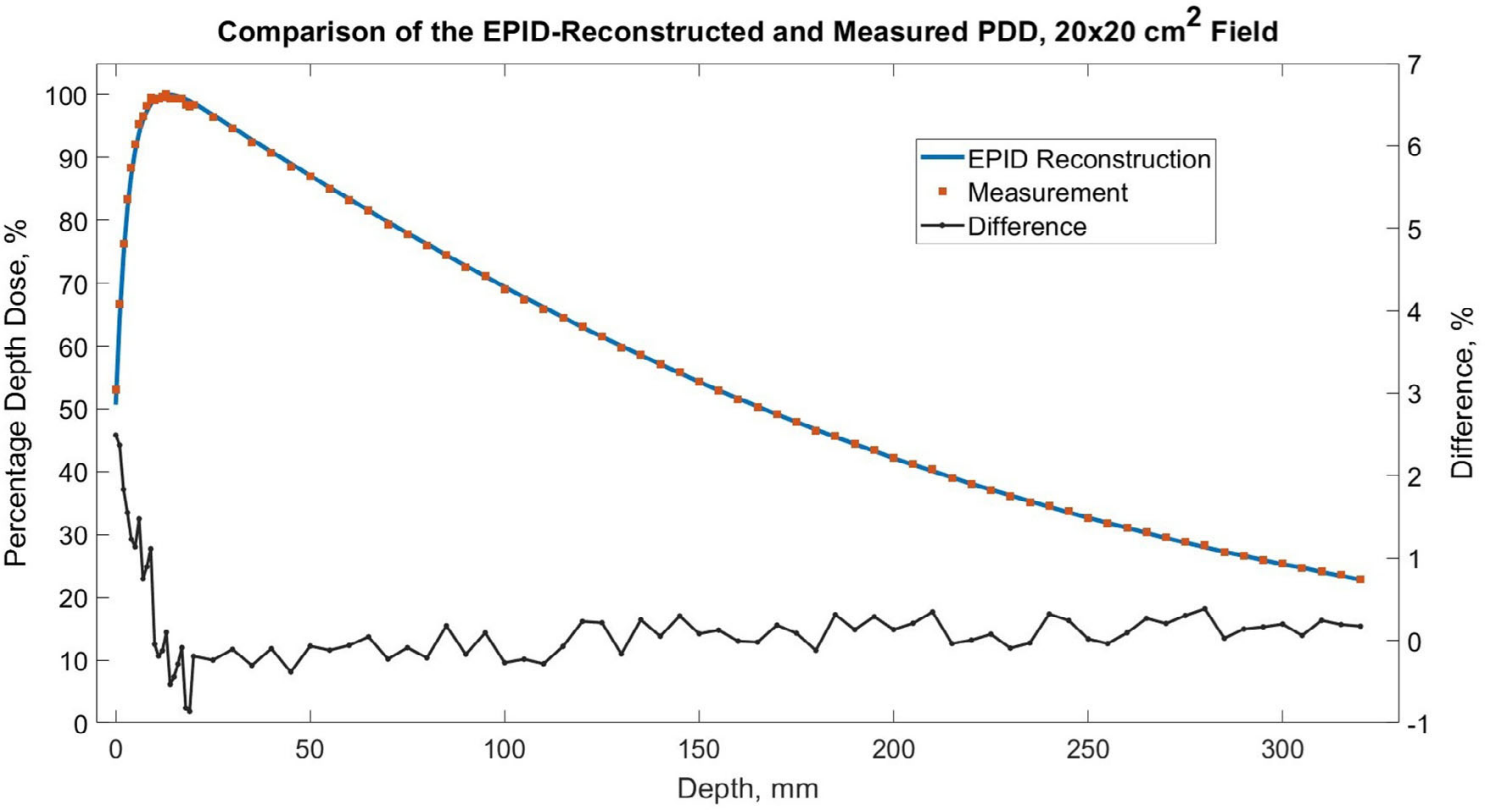
Comparison of EPID‐reconstructed and water tank‐measured PDD curves for 20 × 20 cm^2^ field size.

Figure 2 demonstrates the comparison of EPID‐reconstructed and water tank‐measured PDD for a 20 × 20 cm^2^ incident field. The EPID‐reconstructed PDD agreed with the reference water tank measurement within 1% beyond the first 10 mm of depth in water and within 3% in the first 10 mm of depth in water, for both field sizes.

**FIGURE 3:**
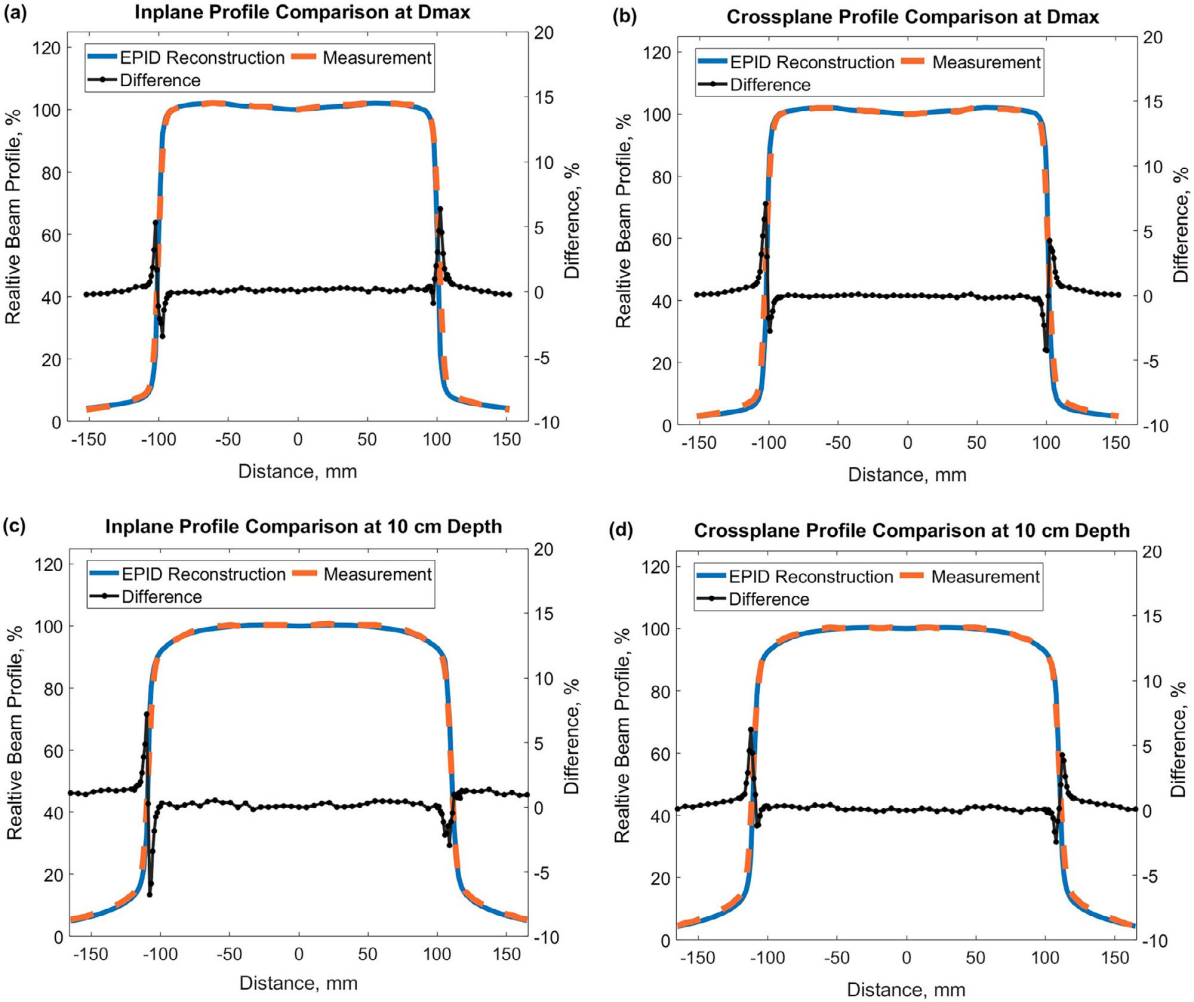
Comparison of EPID‐reconstructed and water measured beam profiles for 20 × 20 cm^2^ field: (a) inplane profiles at d_max_, (b) crossplane profiles at d_max_, (c) inplane profiles at 10 cm depth, and (d) crossplane profiles at 10 cm depth.

Figure 3 shows the comparison of EPID‐reconstructed and water tank‐measured CAX beam profiles for a 20 × 20 cm^2^ field at d_max_ (12 mm) as well as at 10 cm depth. The difference between EPID‐reconstructed profiles and reference measurements is within 1% in low dose‐gradient regions for all profiles. Comparisons of PDD and profiles for a 10 × 10 cm^2^ field at d_max_ (12 mm) and 10 cm depth were also within 1% (not shown).

Table 1 summarizes and compares beam flatness and symmetry values calculated for all the beam profiles presented in Figure 3 and also for the 10 × 10 cm^2^ incident field. The differences between the values based on EPID‐reconstructed dose distributions and the values based on scanning water tank measurements are within 0.2% for all measurements at d_max_ and within 0.3% for all measurements at 10 cm depth.

**TABLE 1:** Comparison of beam flatness and symmetry values calculated using EPID‐reconstructed beam profiles (i.e., virtual water tank) and corresponding water tank‐measured beam profiles.

Field size	Depth in water	Beam parameter	EPID reconstruction	Tank measurement	Difference
10 × 10 cm^2^	d_max_, 14 mm	Inplane flatness	0.59 %	0.67 %	−0.08 %
		Inplane symmetry	−0.42 %	−0.61 %	0.19 %
		Crossplane flatness	0.81 %	0.96 %	−0.15 %
		Crossplane symmetry	0.73 %	0.56 %	0.17 %
	10 cm	Inplane flatness	2.53 %	2.86 %	−0.27%
		Inplane symmetry	−0.58 %	−0.83 %	0.25 %
		Crossplane flatness	2.38 %	2.56 %	−0.18 %
		Crossplane symmetry	0.45 %	0.56 %	−0.11%
20 × 20 cm^2^	d_max_, 12 mm	Inplane flatness	1.13 %	1.23 %	−0.10 %
		Inplane symmetry	−0.41 %	−0.58 %	0.17 %
		Crossplane flatness	1.05 %	0.96 %	0.09 %
		Crossplane symmetry	0.47 %	0.63 %	−0.16 %
	10 cm	Inplane flatness	2.30 %	2.54 %	−0.24 %
		Inplane symmetry	−0.79 %	−0.94 %	−0.15 %
		Crossplane flatness	2.15 %	2.06 %	0.09 %
		Crossplane symmetry	0.57 %	0.78 %	−0.21 %

### 3.2 Assessment of model sensitivity using asymmetrical open fields

**FIGURE 4:**
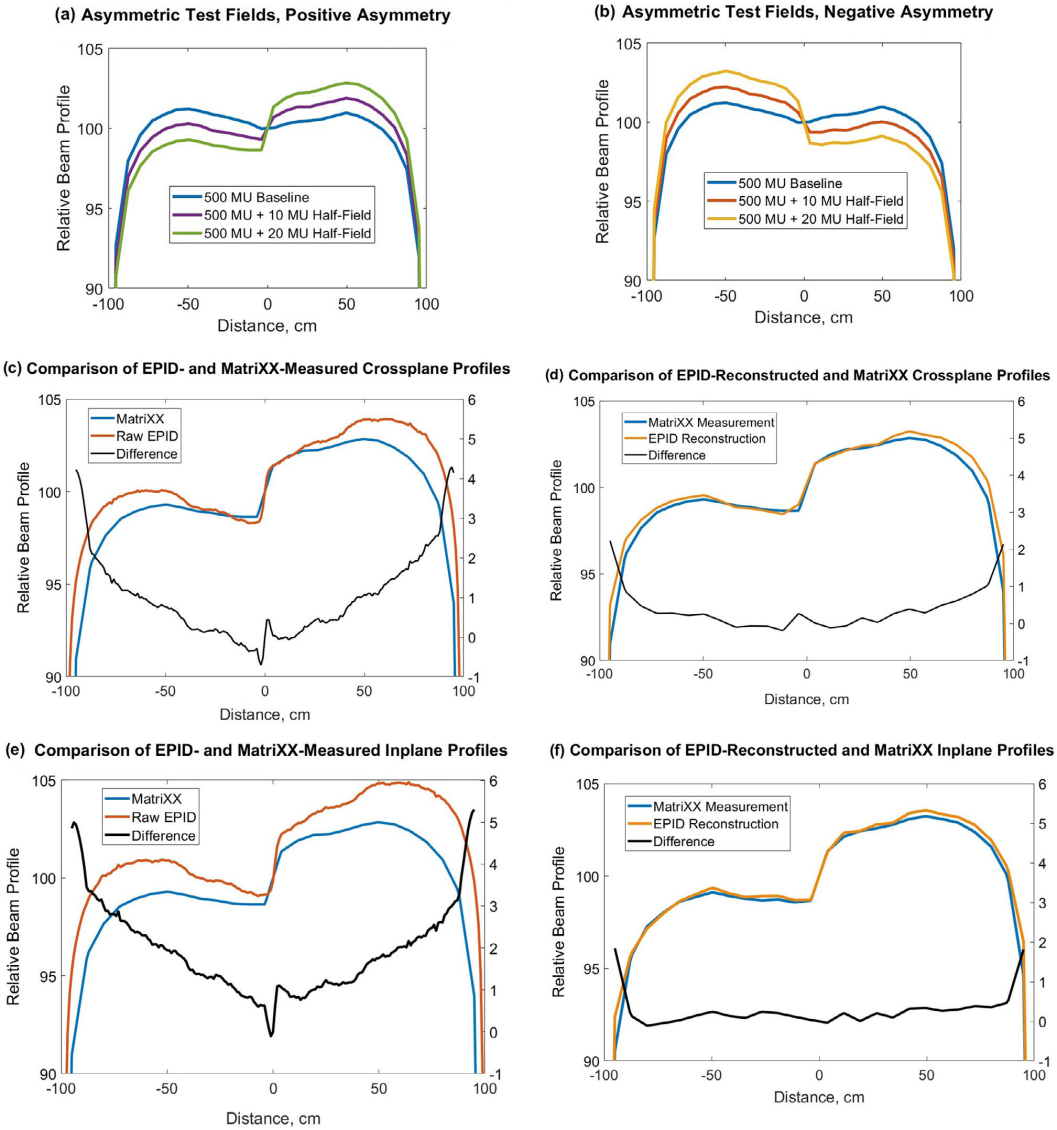
(a, b) MatriXX‐measured crossplane beam profiles for 20 × 20 cm^2^ asymmetric fields: (a) Positive asymmetry. (b) Negative asymmetry. (c–f) Comparison of EPID‐measured and EPID‐reconstructed water‐equivalent profiles against reference MatriXX measurements in crossplane direction: (c) and (d), and inplane direction: (e) and (f).

Figure 4a,b show examples of the MatriXX‐measured asymmetric fields (crossplane profiles) that were used to test the model sensitivity to incident fluence distortions. Figure 4c–f demonstrate a comparison of the “raw” EPID‐measured profiles and EPID‐reconstructed water‐equivalent profiles (i.e., virtual MatriXX) against the reference MatriXX measurement for two fields with the maximum introduced distortion in both crossplane (Figure 4c,d) and inplane (Figure 4e,f) directions. EPID‐reconstructed water‐equivalent profiles show significantly better comparison with the MatriXX measurements than the raw EPID images.

Table 2 compares beam flatness and symmetry values calculated from the raw EPID profiles, EPID‐reconstructed water‐equivalent profiles (i.e., virtual MatriXX), and measured MatriXX profiles, for all test fields used.

**TABLE 2:** Beam flatness and symmetry based on EPID measurements, water‐equivalent EPID reconstruction (WE‐EPID) and reference MatriXX measurements.

Asymmetry direction and level			Negative asymmetry				Baseline	Positive asymmetry			
			−4%	−3%	−2%	−1%	0%	1%	2%	3%	4%
Inplane	Symmetry	Raw EPID	−4.62	−3.44	−2.24	−0.99	0.27	1.75	2.96	4.19	5.38
		WE‐EPID	−3.59	−2.539	−1.549	−0.76	0.21	1.54	2.41	3.32	4.16
		MatriXX	−3.78	−2.69	−1.65	−0.55	0.42	1.78	2.63	3.43	4.33
	Flatness	Raw EPID	2.95	2.53	2.08	1.64	1.23	1.59	2.03	2.46	2.92
		WE‐EPID	2.76	2.38	1.95	1.51	1.19	1.66	2.16	2.62	3.12
		MatriXX	2.61	2.24	1.85	1.43	1.09	1.75	2.23	2.72	3.22
Crossplane	Symmetry	Raw EPID	−4.90	−3.78	−2.63	−1.44	−0.24	1.18	2.32	3.491	4.63
		WE‐EPID	−4.54	−3.60	−2.33	−1.35	−0.42	1.13	1.58	2.84	3.36
		MatriXX	−4.39	−3.40	−2.45	−1.45	−0.57	0.85	1.75	2.60	3.55
	Flatness	Raw EPID	2.84	2.43	1.99	1.56	1.17	1.53	1.95	2.37	2.81
		WE‐EPID	3.04	2.59	2.11	1.64	1.11	1.25	1.79	2.24	2.70
		MatriXX	3.25	2.75	2.22	1.70	1.07	1.17	1.64	2.12	2.594

**FIGURE 5:**
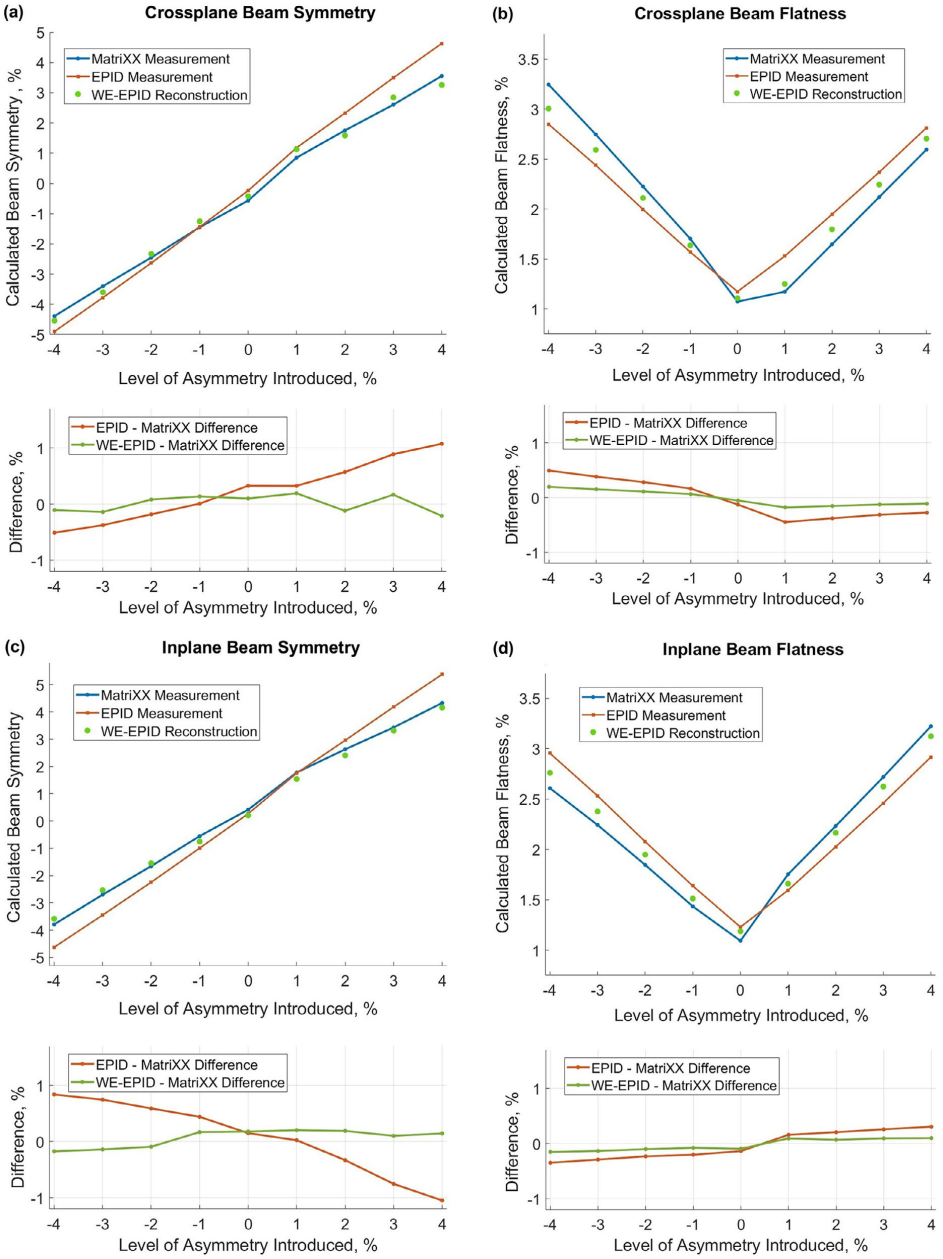
Crossplane (a, b) and inplane (c, d) values of flatness and symmetry determined by all methods: EPID measurement, water‐equivalent EPID reconstruction, and reference MatriXX measurement.

For both inplane and crossplane measurements, the difference between the symmetry determined from the raw EPID and the MatriXX is within 1.1%, while the difference between the symmetry of the water‐equivalent EPID reconstruction and the MatriXX is within only 0.3%. For both inplane and crossplane measurements, the difference between the flatness determined from the raw EPID images and the MatriXX measurement is within 0.4%, while the difference in flatness determined from the water‐equivalent EPID reconstruction and MatriXX measurement is within only 0.2%. Figure 5 shows data presented in Table 2 and visually demonstrates the improvement in the accuracy of determination of beam flatness and symmetry when using the water‐equivalent EPID reconstruction compared to raw EPID images.

### 3.3 Validation of model accuracy using the controlled beam de‐steering

Table 3 demonstrates a comparison of the detuned beam parameters determined by different methods. Beam flatness and symmetry determined based on raw EPID measurements and water‐equivalent EPID reconstruction are compared against the reference MatriXX measurements, and the deviations are calculated. The beam parameters derived from the EPID water‐equivalent reconstruction values are all much closer to those derived from the MatriXX measurements than the values derived from the raw EPID measurements.

**TABLE 3:** Beam flatness and symmetry of detuned beams. Data are based on EPID measurements, water‐equivalent EPID reconstruction (WE‐EPID) and reference MatriXX measurements. Deviation of each EPID‐based method from the reference MatriXX measurements is indicated.

Field type	Beam parameter	Reference MatriXX	Raw EPID	Deviation	WE‐EPID	Deviation
1. Baseline	Inplane flatness	0.97%	1.12%	0.15%	1.08%	0.11%
	Inplane symmetry	0.28%	0.45%	0.17%	0.43%	0.13%
	Crossplane flatness	1.04%	1.18%	0.14%	1.16%	0.12%
	Crossplane symmetry	−0.47%	−0.14%	0.33%	−0.29%	0.18%
2. Positive crossplane detune	Crossplane flatness	1.89%	2.36%	0.47%	2.05%	0.16%
	Crossplane symmetry	2.11%	2.88%	0.77%	2.39%	0.28%
3. Negative crossplane detune	Crossplane flatness	2.13%	2.51%	0.38%	2.30%	0.17%
	Crossplane symmetry	−2.82%	−3.74%	−0.92%	−3.04%	−0.23%
4. Negative inplane detune	Inplane flatness	2.18%	2.60%	0.42%	2.36%	0.18%
	Inplane symmetry	−2.76%	−3.62%	−0.86%	−3.01%	−0.25%
5. Positive inplane detune	Inplane flatness	2.62%	2.99%	0.37%	2.81%	0.19%
	Inplane symmetry	3.49%	4.56%	1.07%	3.76%	0.27%
6. Both crossplane and inplane detune	Inplane flatness	2.48%	2.91%	0.43%	2.63%	0.15%
	Inplane symmetry	−3.23%	−4.32%	−1.09%	−3.47%	−0.24%
	Crossplane flatness	2.27%	2.59%	0.32%	2.13%	−0.14%
	Crossplane symmetry	3.14%	4.17%	1.03%	2.98%	0.16%

**FIGURE 6:**
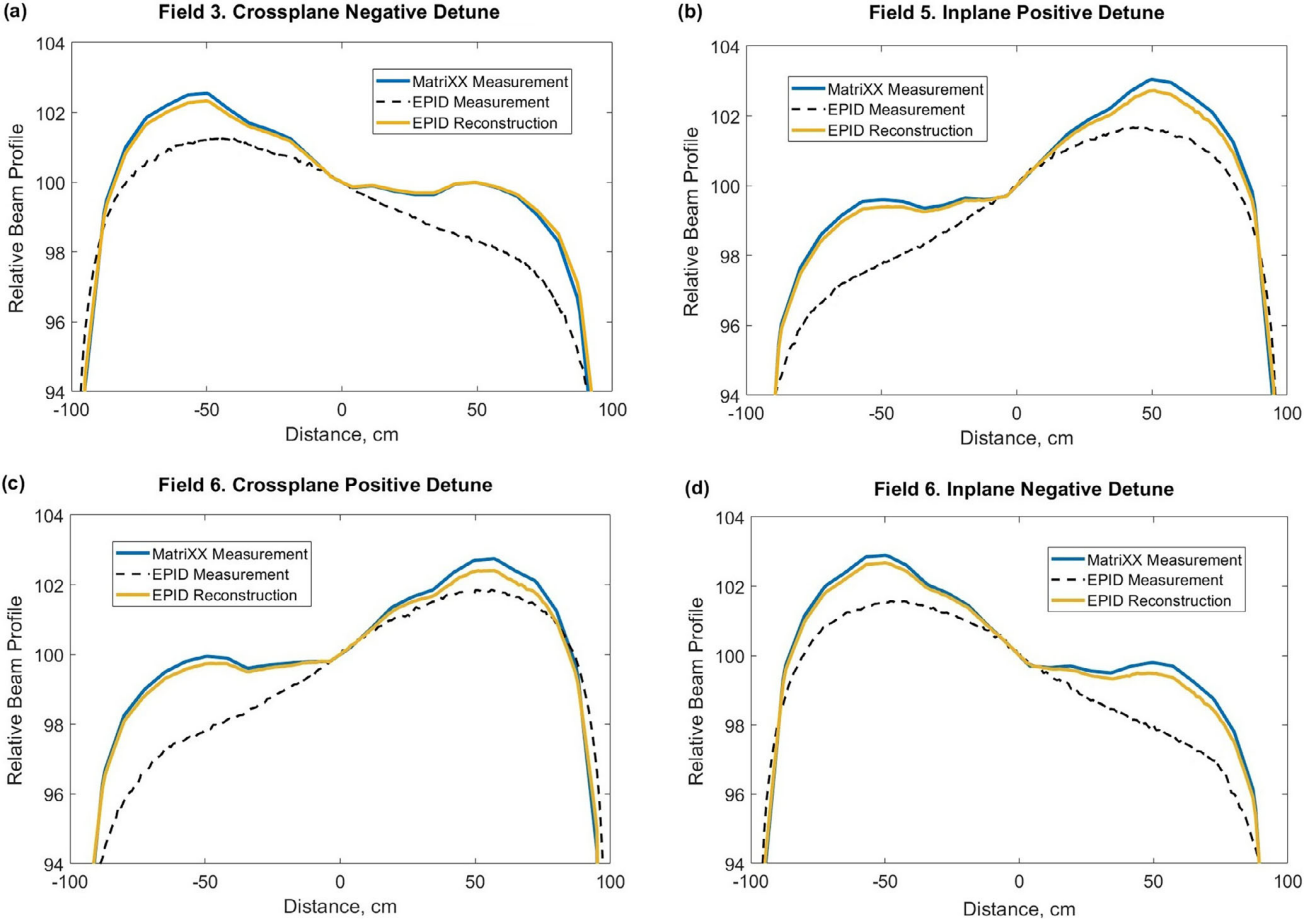
Comparison of MatriXX‐measured, raw EPID‐measured, and EPID‐reconstructed CAX profiles of detuned beams.

Figure 6 demonstrates an example comparison of some of the profiles from Table 3. In particular, it shows a crossplane profile of Field 3 (Figure 6a), an inplane profile of Field 5 (Figure 6b) and both crossplane and inplane profiles of Field 6 (Figure 6c,d). All figures demonstrate better conformity of the EPID‐reconstructed profiles to the MatriXX‐measured profiles, compared to the raw EPID‐measured profiles.

## 4 DISCUSSION

The comparison of EPID‐reconstructed and water tank‐measured PDD for 20 × 20 cm^2^ incident field (Figure 2) demonstrates close agreement within 1% beyond the first 10 mm of depth in water and within 3% in the first 10 mm of depth in water. The increased discrepancy between the EPID‐reconstruction and measurement at shallow depths is likely due to the absence of contaminant electron modeling in the in‐house developed radiation transport model used in this study, a known limitation of the model^32^, ^33^, ^34^, ^35^, ^36^ and falls within reported uncertainties.^39^

The close agreement between the measured and reconstructed (i.e., virtual) PDDs demonstrates overall accuracy of the head fluence modeling, including the beam spectrum, EPID dose image prediction and iterative fluence reconstruction algorithms, as well as 3D dose calculation in a water tank. The accuracy of all three components combined ensures the overall accuracy of the dose reconstruction in a virtual water phantom. Conversely, if any of the above‐mentioned model components (e.g., beam spectrum) changes, this will negatively affect the overall accuracy of the dose reconstruction. This leads to the possibility of detecting changes in the linac beam quality based on the comparison of the EPID‐reconstructed PDDs against the PDDs measured at the machine commissioning or the most recent QA. The difference between these PDDs might be an indicator of possible changes in the linac beam spectrum. The feasibility of this technique to identify linac beam spectrum changes including accuracy and sensitivity will be further investigated in a separate study. It will involve EPID reconstruction of PDDs for various field sizes, including larger fields (e.g., greater than 20 × 20 cm^2^), and its validation against independent reference measurements in a scanning water tank.

The comparison of EPID‐reconstructed beam profiles with reference water tank measurements demonstrates close agreement for all tested field sizes and depths in water. All beam flatness and symmetry values calculated from the EPID‐reconstructed dose distributions agree with the values derived from the scanning water tank measurements to within 0.2% at d_max_ and to within 0.3% at 10 cm depth (Table 1). The proposed EPID image conversion method to a virtual water phantom can calculate corresponding dose profiles in water at arbitrary depths based on measured nonwater‐equivalent EPID images. These profiles can be used to accurately estimate beam flatness and symmetry in water, based on raw EPID images. The method uses robust comprehensive fluence modeling, demonstrates high accuracy, and can be used as a supplementary QA technique in addition to independent reference water tank measurements.

The experiment with intentionally introduced asymmetric fields was conducted to evaluate the sensitivity of the proposed model‐based algorithm to small incident fluence distortions. Generic asymmetric fields used in this experiment demonstrated incrementally changing beam symmetry, confirmed with measurement by a water‐equivalent MatriXX detector. The results show that the algorithm can detect and accurately quantify even small incident fluence distortions. In particular, it was found that the water‐equivalent conversion significantly improves agreement between EPID‐derived and MatriXX‐measured beam profiles (Figure 4c–f). Both inplane and crossplane EPID‐reconstructed profiles agreed with the reference MatriXX measurements to within 1% in the inner 80% of beam width used to determine beam flatness and symmetry. At the same time, the corresponding difference between the raw EPID profiles and MatriXX profiles may reach up to 3.5% due to the differential off‐axis response of a‐Si EPID detectors previously described in literature.^38^

Another observation from the asymmetric fields experiment is that raw EPID measurements tend to overestimate the absolute value of beam symmetry compared to water‐equivalent MatriXX profiles. The observed discrepancy increases with the increased level of the incident beam asymmetry (Figure 5, Table 2). Also, raw EPID measurements may either overestimate or underestimate beam flatness compared to reference MatriXX measurements. Water‐equivalent image conversion significantly improves the accuracy of derivation of both these parameters, as is demonstrated in Table 2 and Figure 5.

The experiment with detuned beams was conducted to simulate beam steering issues (e.g., due to steering magnets malfunction) and to evaluate the sensitivity of the image conversion algorithm to beam non‐uniformities caused by incorrect beam steering. Similar experiments have been conducted by other research groups,^13^, ^15^ although without water‐equivalent EPID image conversion. For example, Sun et al. used detuned beams to assess the possibility of detecting beam flatness and symmetry changes using raw EPID images.^13^ While their method was able to detect such changes, it usually overestimated the magnitude of measured beam symmetry by up to 1.25% and either over‐ or underestimated beam flatness by up to 0.9% compared to reference MatriXX measurements.^13^

The algorithm proposed in this study does not only detect beam flatness and symmetry changes, but also much more accurately quantifies these changes in a water‐equivalent manner. In the detuned beams experiment, similarly to the publication by Sun et al.,^13^ raw EPID measurements overestimated beam symmetry by up to 1.1% compared to reference MatriXX measurements. At the same time, the agreement of raw EPID‐calculated beam flatness with MatriXX was within 0.5% for all test fields (Table 3). The application of the water‐equivalent conversion algorithm reduced these deviations to within 0.3% and 0.2% for symmetry and flatness values, respectively (Table 3). In addition, it significantly improved conformity of all EPID‐measured profiles to the reference MatriXX‐measured profiles (Figures 4 and 6).

Today linac manufacturers are introducing integrated image‐based QA tools for daily verification of linac beam and mechanical performance.^1^ One such example is the Varian Machine Performance Check (MPC). MPC is a fully integrated tool for daily assessment of machine output, beam center, profile uniformity, and several geometric and mechanical linac parameters against user‐defined baselines.^15^ Hence, several groups have proposed comprehensive linac QA applications that use EPID images.^12^, ^13^ These applications currently rely on uncorrected, nonwater‐equivalent EPID images, which limits their applications to constancy checks relative to baselines.^15^, ^17^ Users would benefit if tools like MPC were able to check more radiation beam parameters (e.g., beam flatness and symmetry, or beam quality). The application proposed in this study performs water‐equivalent EPID image conversion, which leads to more accurate determination of beam flatness and symmetry values. This application could be integrated into vendor‐provided EPID‐based QA tools to provide users with the accurately estimated water‐equivalent beam flatness and symmetry values on a daily basis, in addition to routine machine output and profile constancy checks.

Future work will focus on expanding and validating the method's applicability for detecting changes in beam quality and further investigating its utility for comprehensive linac QA application. While the current work focuses on validating the developed algorithm for flattened 6MV beams only, in future work, its water‐equivalent image conversion functionality will be extended and validated for high‐energy beams (e.g., 10MV) and flattening filter free (FFF) beams.

## 5 CONCLUSION

Amorphous silicon EPID detectors demonstrate many favorable dosimetric parameters. However, they have a nonwater‐equivalent energy response, which is an obstacle to some dosimetric applications. In particular, absolute beam flatness and symmetry cannot be determined using EPID measurements, which to date have limited EPID applications to relative constancy checks. EPID‐to‐water image conversion algorithms exist, but most are correction‐based and have not been applied to linac dosimetry applications. Simple correction‐based approaches utilize empirical correction functions and lack robustness and accuracy. Model‐based image conversion algorithms are not extensively studied in literature despite having superior accuracy, robustness, and flexibility.

This study presents a novel EPID‐based machine QA application that employs a previously developed model‐based radiation transport algorithm to convert EPID‐measured images into two‐ or three‐dimensional water‐equivalent dose distributions (i.e., virtual water phantom). The proposed method enables accurate determination of linac beam parameters, including beam flatness, symmetry, and, potentially, beam quality, thus addressing some limitations of previous EPID‐based techniques restricted to constancy checks.

Validation against independent reference measurements in a scanning water tank and ion chamber array demonstrates high accuracy of the proposed algorithm, with EPID‐reconstructed PDDs agreeing to within 1% beyond the first 10 mm depth, and beam profile comparisons also showing agreement within 1% in low dose‐gradient regions. Furthermore, model sensitivity assessments with controlled beam asymmetry and beam de‐steering experiments confirm the robustness of the proposed method, with significant improvements in accurately determining beam flatness and symmetry (within 0.2% and 0.3%, respectively) compared to raw EPID images (within 0.5% and 1.1% for beam flatness and symmetry, respectively).

## AUTHOR CONTRIBUTIONS

Ivan Kutuzov: Conceived and designed the idea of the experiment. Developed and implemented MATLAB code to integrate the dose prediction and fluence reconstruction algorithms for EPID image conversion into water‐equivalent dose distributions. Performed measurements using EPID, 3D scanning water tank, and ion chamber array. Conducted dose reconstruction from EPID images, analyzed and compared results with reference measurements. Wrote and reviewed the manuscript. Boyd McCurdy: Conceived and designed the idea of the experiment. Provided overall guidance and leadership of the experiment through a series of reviews and discussions. Reviewed the methods and results of the experiment. Reviewed the manuscript.

## CONFLICT OF INTEREST STATEMENT

The authors declare no conflicts of interest.

## ETHICS STATEMENT

This study did not involve any human participants or animals. Therefore, ethical approval and informed consent were not required, and the authors have no ethical disclosures to report.
